# Massive gas embolism secondary in the use of intraoperative hydrogen peroxide: still use to lavage with this liquid?

**DOI:** 10.11604/pamj.2013.16.124.3499

**Published:** 2013-11-29

**Authors:** Zine El Abidine Benali, Hatim Abdedaim, Driss Omari

**Affiliations:** 1 Department of Anesthesiology & Intensive Care, CHP Eddarak, BERKANE, Morocco; 2 Department of Anesthesiology & Intensive Care, Military Hospital Mohammed V, University Mohammed V Souissi, Rabat, Morocco; 3 Department of Internal Medicine and Cardiovascular Diseases, CHP Eddarak, Berkane, Morocco

**Keywords:** Gas embolism, hydrogen peroxide, lavage, surgery, operation

## Abstract

Cases of embolism after using hydrogen peroxide have been described in many circumstances in the operating room. Hydrogen peroxide is not more effective than other antiseptics; its potentially serious risk should not be unrecognized. The alternative use of saline seems very reasonable. The widespread use of hydrogen peroxide by practitioners is explained mainly by its antiseptic effect associated with effervescent backlash visual and auditory, but sometimes the liquid hiding behind a black hole that absorbs the life of the patient in case of inappropriate use. Diagnosis is based on clinical variations in a conscious patient at the time of use, confirmed by echocardiology if available. We related the case of a massive embolism after hydrogen peroxide use in the cleaning of infected wound with osteosynthesis material left femoral done under spinal anesthesia in a young girl of 17 years admitted after to the ICU intubated ventilated.

## Introduction

Hydrogen peroxide (H2O2) is widely used for cleansing and dressing of surgical wounds, partly due to tradition, partly due to its pleasing visual effect. If O2 bubbles produced after catalysis of H2O2 diffuse into the venous circulation, it may be responsible for a morbidity or even mortality. Surgeons, the first users of H2O2 seem poorly informed about the dangers of certain practices using this molecule. We report a case of massive gas embolism in a patient secondary in the use of intraoperative hydrogen peroxide for cleaning of infected wound and osteosynthesis material on the left femoral.

## Patient and observation

**Figure 1:**
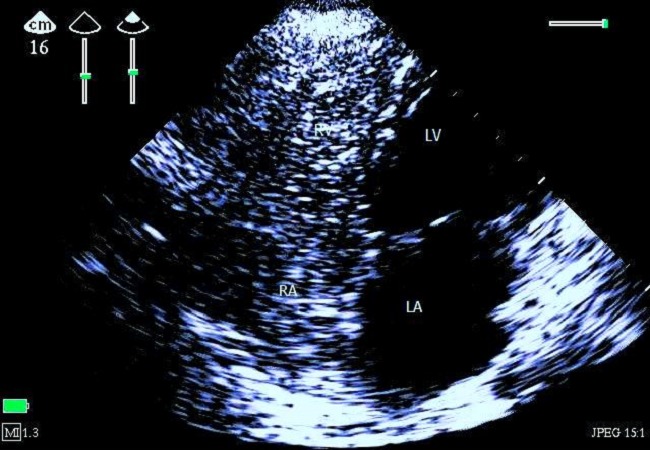
Apical four-chamber 2D echocardiography centered on the right cavities showing massive gas bubbles, image taken 50 seconds after the onset of clinical signs of embolism. (LA: left atrium, LV: left ventricle, RV: right ventricle, RA: right atrium)

This is a young woman of 17 years with no history pathological individuals, operated five days earlier in the same hospital for fracture of the femoral shaft with introduction of osteosynthesis material, three days after the patient had an infection of the operative quote with pus necessitating washing and antibiotics, the first cleaning with H2O2 (low volume of 20 ml) was done in the service of surgery and the trauma observed a transitory dyspnea during use this solution H2O2. Two days after, the second clean was done in the operating room, pre-anesthetic examination showed a patient in good general condition, surgical wound infection, and skin examination did not show vascular malformations including hemangiomas. The cardiovascular, pulmonary examination, biological and pulmonary radiology were normal, it was decided to make a spinal anesthesia after a filling of 500 cc of saline to ease the surgeon′s work for a good clean and check of osteosynthesis material, this with a standard monitoring: oxygen saturation (SpO2), scope, and blood pressure. Two min after using 350 ml H2O2, the patient presented stirring with chest tightness followed by loss of consciousness, blood pressure dropped from 120/ 75 mm hg to 75/35 mm hg, SpO2 decreased abruptly to about 94%. Cardiac auscultation revealed a noise wheel (sound metallic) but was strictly normal lung especially in sloping areas, the scope showed a tachycardia of 92 beat / min but without ST segment change. Considering the context of use H2O2, the massive gas embolism was very suspicious. asked to immediately stop the surgical gesture, restored blood pressure with titration of epinephrine, the patient was intubated ventilated without drugs with FIO2 100%, and she was Placed in the Trendelenburg position. A Portable echocardiography showed gas bubble in the right cavities, and were dilated, confirming the massive gas embolism (Figure 1), and also showed a 1 cm diameter of the vena cava inferior in faveure of hypovolemia still required rapid filling, a central venous right internal jugular ultrasound guided was set for emergency suction bubble but unfortunately it did not bring a lot of gas, the patient was transferred to the intensive care unit, placed under mechanical ventilation with vasoactive drugs for ten hours and then extubated without neurological sequelae, other cleaning with saline after the incidence of embolism were performed without problems. Further research oval foramen and intrapulmonary shunt was negative.

## Discussion

Repeated description of these accidents [1] to H2O2 and the low level of evidence of its superiority over other antiseptic agents raise the issue of its use today, used this bactericidal solution yes or not?. Hydrogen peroxide used them at risk of gas embolism in case of incorrect use among hypovolemic patients. Gaseous emboli result from a direct passage of gaseous oxygen into the systemic circulation by intra cavitary hyper pressure oxygen or intravascular absorption of hydrogen peroxide with secondary formation of bubbles. H2O2 is decomposed by many substances, including blood which promotes its catalysis according to the reaction [2]: 2H2O2 → 2H2O + O2, and O2 release effervescent bubbles have a bactericidal and bacteriostatic effect, mainly for anaerobes germs. On knowing their oxygen release is important because each 1 ml of hydrogen peroxide is capable of releasing 10ml of oxygen [3]. These complications are seen especially in cases: the use of large volumes (sometimes even low volume), injection with pressure, and the tissue dilapidated irrigation with significant venous wounds or closed cavities especially in the context of hypovolemia [4, 5].

Common clinical signs include the sign of the wheel perceived on auscultation in cases of massive embolism as in our case report, a sharp drop capnia associated with a decrease in SpO2 secondary the intrapulmonary shunt and hemodynamic disorders or cardiogenic shock by right ventricular dysfunction [6], neurological disorders are possible and can be explained either by decreased cardiac output, either directly by a paradoxical embolism in the case of a patent foramen ovale or rarely by intrapulmonary shunt with Overtake the purification and passing gas bubble in the systemic circulation, in our patient the agitation and chest tightness (conscious patient under spinal anesthesia) we drew attention in the context of use of oxygenated water and immediately asked to stop irrigation. The diagnosis is reported by using portable echocardiography in the operating room 50 seconds after the event has shown that the massive gas bubble in the right cavities during agitation. The most recommended treatment was consistent with the following recommendations: stop the cause of embolism, ventilate with FiO2 100% and put in the Trendelenburg position, in other cases, complete surgical site with saline or wet compresses [6]. Hyperbaric oxygen therapy is indicated based on the clinical picture to include the presence of severe neurological signs including deficit [7].

Morbidity and mortality from oxygen liberation has been reported ever since the early use of hydrogen peroxide in the 1850s [8]. The use of hydrogen peroxide as an antiseptic has no direct benefits, but is associated with significant risk, therefore informed the practitioners especially surgeons for risque utilization H2O2. There is however, good evidence that high volume, medium or low pressure pulsed saline lavage is safe and powerful at reducing bacterial loads and removing wound debris [9, 10]. In fact if any additive substance used with saline lavage has been demonstrated to be effective, from wounds it is the humble soap [10].

## Conclusion

The risk-benefit balance does not appear for the indication of lavage with Hydrogen peroxide. An alternative such as saline solution with or without soap seems reasonable. Prevent the anesthetist when using Hydrogen peroxide before irrigation in all surgeries, use a cup instead of a syringe to avoid pressure injection, use a low volume and outlaw its use in hollow cavities richly vascularized in the context of hypovolemia.
